# Porcine Mx1 fused to HIV Tat protein transduction domain (PTD) inhibits classical swine fever virus infection in vitro and in vivo

**DOI:** 10.1186/s12917-015-0577-4

**Published:** 2015-10-15

**Authors:** Xiaomin Zhang, Jiao Jing, Wenliang Li, Ke Liu, Baojun Shi, Qianqian Xu, Zhiyong Ma, Bin Zhou, Puyan Chen

**Affiliations:** College of Veterinary Medicine, Nanjing Agricultural University, Nanjing, 210095 China; Institute of Veterinary Medicine, Jiangsu Academy of Agricultural Sciences, Nanjing, 210014 China; Shanghai Veterinary Research Institute, Chinese Academy of Agricultural Science, Shanghai, 200241 China

**Keywords:** Porcine Mx1 fused to HIV Tat-PTD (PTD-poMx1), Classical swine fever virus (CSFV), Antiviral activity, in vitro, in vivo

## Abstract

**Background:**

Classical swine fever (CSF) caused by CSF virus (CSFV) is highly contagious andcauses significant economic losses in the pig industry throughout the world. Previously we demonstrated that porcine Mx1 (poMx1), when fused to HIV Tat protein transduction domain (PTD), inhibits CSFV propagation in PK-15 cells, but it is unknown whether PTD-poMx1 exhibits antiviral activity in other porcine lines and it is efficacious for controlling CSFV infection in pigs in China.

**Methods:**

Two porcine cell lines, ST and 3D4/21, were used to investigate in vitro antiviral activity of PTD-poMx1 against CSFV using confocal microscopy, western blot, flow cytometry, and real-time RT-PCR. Furthermore, in vivo antiviral activity of PTD-poMx1 was assessed by means of rectal temperature, clinical score, pathological lesion, white blood cell count, viral load, etc.

**Results:**

PTD-poMx1 entered both cell lines within 3 h and maintained for 16 h, but did not affect CSFV binding and uptake. Viral titers and qRT-PCR data showed that PTD-poMx1 inhibited CSFV replication in both cell lines, showing significant antiviral activity after infection. Injection of PTD-poMx1 into CSFV-challenged pigs attenuated CSFV symptoms and viremia in dose-dependent manner but did not completely block virus replication within 14 days post challenge, suggesting that PTD-poMx1 confers partial protection against a lethal challenge.

**Conclusion:**

We demonstrated the anti-CSFV activity of PTD-poMx1 in vitro and in vivo. The results have shown that treatment with PTD-poMx1 alleviated symptoms and viral load in infected pigs. The results support our previous in vitro studies and suggest that PTD-poMx1 could be promising in reducing the clinical signs caused by CSFV.

## Background

Classical swine fever (CSF) is caused by CSF virus (CSFV), a small, enveloped, positive-strand RNA virus of the genus *Pestivirus* within the family *Flaviviridae* [1, 2]. CSF is acute and highly contagious in swine and is responsible for severe economic losses in pig production worldwide [3]. Although live attenuated vaccines, including C strain, are still used to inoculate animals for CSF control [4], the inability to serologically differentiate vaccinated from infected pigs has resulted in the ban of prophylactic vaccination in the European Union (EU) [5]. The CP7_E2alf marker vaccine, a *pestivirus* chimera, may be a suitable substitute for controlling CSF outbreaks along with improved diagnostic tools; but the complete protection and immune response conferred by this vaccine needs further study in domestic pigs and *sus scrofa* [6–8]. Because vaccination alone has not been sufficient to control CSF, novel antiviral agents will supplement current vaccine control strategies [9].

Mx proteins are interferon-induced dynamin-like GTPases that are present in all vertebrates [10–12], and have a broad range of antiviral activities [13, 14]. The full-length porcine Mx1 (poMx1) gene was first isolated from the German Landrace breed [15] and mapped to chromosome 13 [16]. Functional poMx1 is located in the cytoplasm of target cells and exhibits antiviral activity against some RNA viruses. Previous studies showed that poMx1 confers resistance to vesicular stomatitis virus [17, 18] and influenza virus [19, 20], suggesting that poMx1 is an important determinant of the IFN-induced antiviral activity. In earlier studies, we noted that EGFP-poMx1 fusion protein overexpressing in PK-15 cells had anti-CSFV activity, and also reported that poMx1 fused to the protein transduction domain (PTD) of HIV [17] inhibited CSFV replication in a dose-dependent manner [21]. Our previous results showed that PTD-poMx1 expressing in *E.coil* inhibits CSFV propagation in PK-15 cells, suggesting that PTD-poMx1 harbors the preventive effect or the therapeutic effect. However, many details are to be expounded as follows: i) How long the exogenous PTD-poMx1 enters the porcine line. ii) How long it can stay in cells after entering. iii) Whether the receptor on the cell surface can be affected due to the exogenous PTD-poMx1, interfering CSFV binding or uptake. More importantly, which step in CSFV lifecycle is inhibited by PTD-poMx1. To address these questions, porcine cell lines (ST and 3D4/21) were used here to evaluate the antiviral activity of the PTD-poMx1 fusion protein in vitro. In addition, PTD-poMx1 was injected into CSFV-challenged pigs to evaluate the antiviral activity in vivo. Overall, our data demonstrated that PTD-poMx1 has anti-CSFV activity in vitro and in vivo, suggesting the feasibility of a preliminary clinical application of PTD-poMx1 against CSF infection.

## Methods

### Cells, virus and fusion protein

Swine testis cells (ST, CRL-1746) were purchased from ATCC and propagated in Dulbecco’s Modified Eagle’s Medium (DMEM, Gibco) supplemented with 10 % fetal bovine serum (FBS, Invitrogen), 100 U/ml penicillin and 100 μg/ml streptomycin. Porcine alveolar macrophage cells (3D4/21, CRL-2843) were a gift from Dr. Guoqing Shao (Institute of Veterinary Medicine, Jiangsu Academy of Agricultural Sciences) and were propagated in RPMI 1640 Medium supplemented with 10 % FBS, 100 U/ml penicillin, 100 μg/ml streptomycin, and Non-Essential Amino Acids (NEAA, Gibco). CSFV Shimen strain was purchased from China Institute of Veterinary Drugs Control. PTD-poMx1 fusion protein was expressed in *E. coli* and purified, denatured and refolded as previously described [17]. Aliquots of purified protein were stored at −80 °C.

### Confocal microscopy

To determine the protein transduction efficiency, internalization of PTD-poMx1 was assessed by confocal microscopy. Briefly, ST and 3D4/21 cells grown on glass coverslips in 6-well plates were treated with 80 μg/ml PTD-poMx1 for 6, 12 and 24 h. Cells were then fixed with 4 % paraformaldehyde in PBS, and permeabilized with 0.2 % Triton X-100. Cells were incubated with anti-poMx1 mAb (1:500, ab79609, Abcam, USA) as a primary antibody for 1 h at 37 °C. After washing three times with PBS, cells were incubated with FITC-labeled goat anti-mouse IgG (1:200, ab150113, Abcam, USA) as a secondary antibody for 30 min at 37 °C. Cell nuclei were counter stained with 4’, 6-diamidino-2-phenylindole (DAPI). Fluorescent images were acquired by LSM710 confocal laser microscopy (Carl Zeiss Microimaging, Oberkochen, Germany), and processed with Photoshop software.

### Western blot analysis

The transduction efficiency and stability of PTD-poMx1 in ST and 3D4/21 cell lines were evaluated by Western blot analysis as described previously [17]. Briefly, cells were cultured as exponentially growing subconfluent monolayers on 6-well plates. 80 μg/ml PTD-poMx1 was added to the cells at 37 °C. Every hour from 1 to 6 h, cells were washed three times with PBS and lysed in cold lysis buffer (1 % Triton X-100, 1 mM PMSF in PBS) for 10 min. Lysates were clarified by centrifugation at 12,000 x g for 10 min. Total cell extracts were separated by SDS-PAGE and transferred onto nitrocellulose membrane. PTD-poMx1 that translocated through the plasma membrane [22] and accumulated in the cytoplasm [17] was detected by anti-poMx1 mAb (1:1000). To assess the stability of PTD-poMx1 in the cells, 80 μg/ml PTD-poMx1 was added to the cells for 6 h at 37 °C. After washing three times with PBS, cells in DMEM free from PTD-poMx1 were incubated for a further 4, 8, 12, 16, and 20 h. Intracellular PTD-poMx1 in the cell lysates was detected by anti-poMx1 mAb (1:1000) as previously described. β-actin, a loading control, was detected by anti-β-actin antibody (1:5000, No. A5441, Sigma-Aldrich, USA).

### Flow cytometry

To determine if PTD-poMx1 affects CSFV entry into host cells, CSFV adsorption at the cell surface was examined after treatment with PTD-poMx1 using flow cytometry and the data were analyzed with Cell Quest Pro software (Becton Dickinson). Briefly, ST or 3D4/21 cells treated with or without 80 μg/ml PTD-poMx1 were inoculated with CSFV at a MOI of 5 for 1 h on ice. The infected cell suspensions were prepared with PBS containing 0.02 % ethylene-diaminetetraacetic acid (EDTA), and incubated with the anti-E2 monoclonal antibody WH303 (1:100, Veterinary Laboratories Agency, Surrey, UK) for 1 h at 37 °C. After extensive wash, cells were treated with FITC-labeled goat anti-mouse IgG (1:200) at 37 °C for 30 min. Fluorescent signals on the cell surface were examined by a flow cytometer (Becton-Dickinson) and the percentage of positive cells was counted among 3 × 10^4^ cells. Untreated cells were used as a negative control.

### Quantitative real-time RT-PCR (qRT-PCR)

Viral genome replication was measured by qRT-PCR as previously described with modifications [21, 23]. Briefly, viral RNA was extracted from each sample using TRIzol reagent (Invitrogen, CA, USA). RNA pellets were suspended in 20 μl DEPC-treated water and a RT reaction was performed utilizing a RT reaction kit (Takara, Dalian, China). Target primers for the NS5B gene [24] and reference primers for the GAPDH gene [25] were used to quantify CSFV RNA. qPCR was carried out with SYBR Green PCR master mix according to the manufacturer’s protocol (Takara, Dalian, China). The data were analyzed by the 2^-△△Ct^ method, and expression of the target gene was normalized to GAPDH mRNA levels in the same samples [26].

### In vitro anti-CSFV activity of PTD-poMx1

Initially, three experiments were performed in parallel to investigate the antiviral activity of PTD-poMx1 against CSFV in ST and 3D4/21 cells. In the first experiment (treatment), cells (1.2 × 10^6^) in 6-well plates were inoculated with CSFV at a MOI of 0.01 for 2 h at 37 °C. Cells were washed three times with PBS and maintained in medium containing 80 μg/ml PTD-poMx1 for 48 h. In the second experiment (prophylaxis), 80 μg/ml PTD-poMx1 was added to the cells (1.2 × 10^6^) for 6 h at 37 °C. Cells were washed three times with PBS, then inoculated with CSFV at a MOI of 0.01 for 2 h at 37 °C. Cells were washed with PBS and maintained in medium free with PTD-poMx1 for 48 h at 37 °C. In the third experiment, 80 μg/ml PTD-poMx1 was inoculated simultaneously with CSFV at a MOI of 0.01 in the cells for 2 h at 37 °C. Cells were washed three times with PBS and maintained in medium free with PTD-poMx1 for 48 h at 37 °C. At 48 h post infection (hpi), viruses within the cells were released by freezing and thawing three times, and qRT-PCR was performed to quantify viral replication.

### Animal experiment design

Nine four-week-old specific-pathogen-free Large White pigs were randomly assigned to three groups of three animals housed in three separate rooms. Prior to experiments, all pigs were examined serologically negative for certain important pathogenic viruses, such as CSFV, PRRSV, PCV2, FMDV, PRV, etc., using a series of commercial ELISA diagnostic kits from IDEXX Laboratories, Inc. and JBT Agency. Furthermore, all pigs were examined pathogenic negative for the tested viruses using a series of PCR/RT-PCR assays (data not shown). All animals were oro-nasally challenged with 10^5^TCID_50_ CSFV Shimen strain. On the first day post challenge (1dpc), pigs in two groups (designated as 1 dose and 3 doses group, respectively) were injected with 1 mg/dose PTD-poMx1 protein via the ear vein. Pigs in the negative control group (designated as NC group) received equivalent saline via the ear vein. Pigs in 3 doses group were injected two additional times with 1 mg PTD-poMx1 at 3 and 5 dpc. Rectal temperature, clinical scores (1 point- no fever; 2 points- pyrexia + mild clinical signs; 3 points- severe clinical signs, and 4 points- death) and pathological lesions were monitored daily as previously described [27]. Blood samples (sera and EDTA-anticoagulated blood) were collected at 0, 3, 5, 7, 11, 14, and 21 dpc. White blood cell (WBC) counts were measured using an Abacus Junior Vet 5 (Diatron Group, Budapest,Hungary). Care of laboratory animals and animal experimentation were performed in accordance with animal ethics guidelines and approved protocols. All animal experiments were approved by the Animal Ethics Committee of Nanjing Agricultural University, and performed in High Technology Innovation Center of Animal Disease Control, Ministry of Agriculture, PR China (BSL-3 condition).

### Viremia detection

Two experiments were performed to evaluate viremia. First, progeny viruses in sera were assessed using a TCID_50_ assay as previously described [28]. Second, viral RNA was extracted from whole blood using the RNeasy mini kit (Qiagen, Courtaboeuf, France), and viral load was detected using qRT-PCR.

### Enzyme-linked immunosorbent assay (ELISA)

Two ELISA assays were performed to detect anti-poMx1 antibody or anti-CSFV-E2 antibody in sera as previously described with some modifications [29]. First, 96-well flat-bottom plates were coated with purified PTD-poMx1 at a concentration of 100 ng/well in coating buffer (Na_2_CO_3_/NaHCO_3_, pH 9.6) at 4 °C overnight. Plates were washed 3 times with PBST, blocked with 1 % BSA in PBST at 37 °C for 2 h, and then washed 3 times with PBST. 100 μl pig sera (1:100) was added to each well. After incubation at 37 °C for 1 h, the plates were washed 3 times with PBST and then incubated with HRP-labeled goat anti-pig IgG antibody (1:5000, sc-2463, Santa Cruz, USA) at 37 °C for 1 h. After the final wash, 100 μl of fresh TMB substrate (Sigma-Aldrich, USA) was added per well and plates were incubated for 10 min. The reaction was stopped by 2 M H_2_SO4, and optical density (OD) was measured at 450 nm using ELISA plate reader (ELX800). Second, the antibodies to CSFV E2 in the sera were assessed using the IDEXX CSFV Antibody Test Kit (IDEXX Laboratories, Inc., Maine, USA) according to the manufacturer’s protocol.

### Statistical Analysis

All data are presented as means ± standard deviation (S.D.) as indicated. Student’s *t*-test was used to compare pairs of treated or untreated groups. Statistical significance is indicated as not significant (ns) (P > 0.05), * (*P* < 0.05), and ** (*P* < 0.01). All statistical analyses and calculations were performed using GraphPad Prism 5 (GraphPad Software Inc, La Jolla, CA).

## Results

### Internalization and stability of PTD-poMx1

**Fig. 1:**
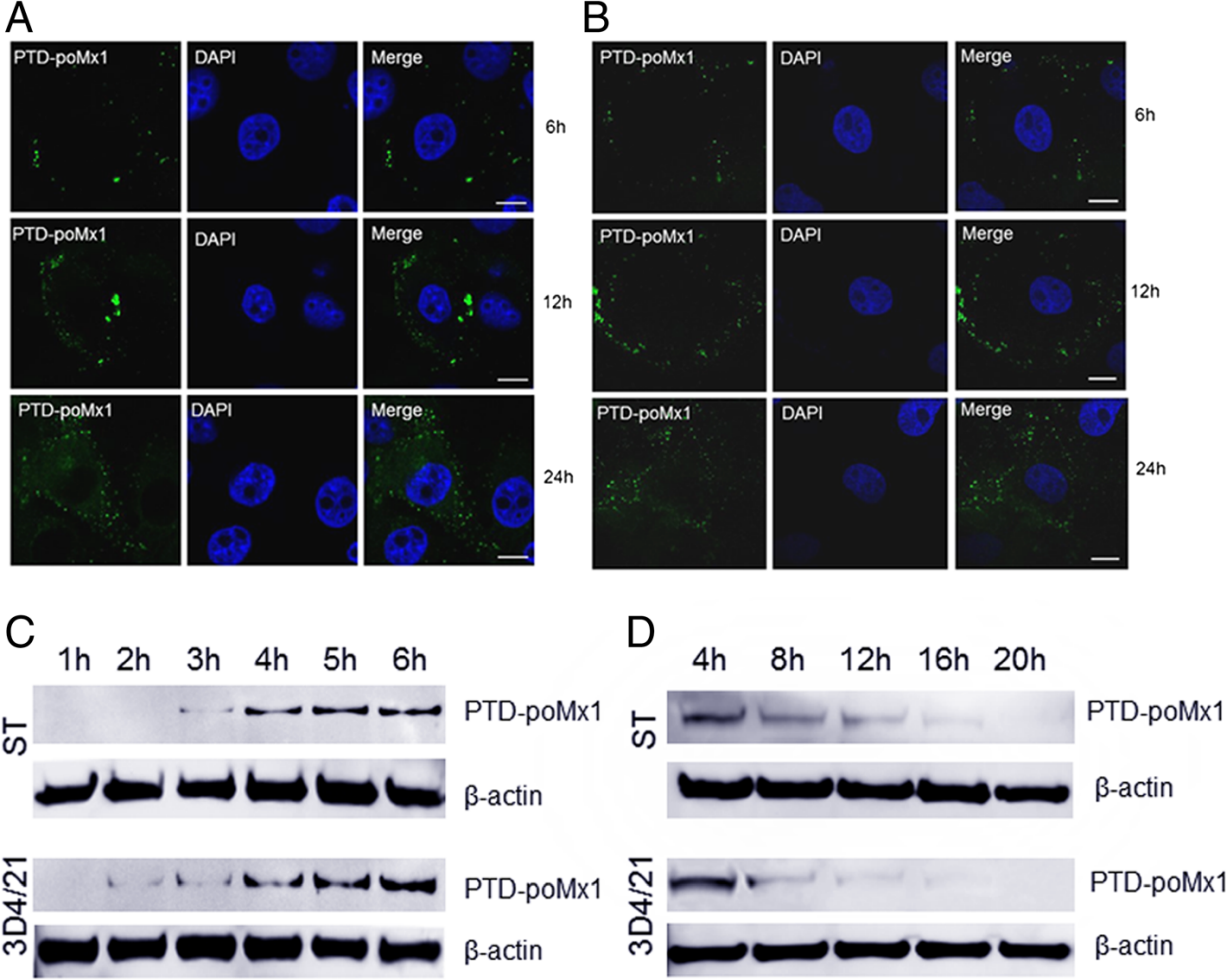
Internalization of PTD-poMx1 fusion protein. Intracellular localization of PTD-poMx1 fusion protein in ST cell line (**a**) and 3D4/21 cell line (**b**) using confocal microscopy. Cells were treated with 80 μg/ml PTD-poMx1 fusion protein for 6, 12 and 24 h at 37 °C, then fixed in 4 % PFA for 15 min at room temperature after three washes with PBS. The anti-poMx1 mAb was applied as a primary antibody for 1 h at 37 °C. After washing three times with PBS, cells were incubated with FITC-goat anti-mouse IgG as a secondary antibody for 30 min at 37 °C. Fluorescent spots attributed to PTD-poMx1 were observed using fluorescence microscopy and recorded with a digital camera. Bar, 10 μm. **c** The transduction kinetics of PTD-poMx1. ST or 3D4/21 cells were treated with 80 μg/ml PTD-poMx1 and sampled every hour for six hours. Each sample was washed three times with PBS and lysed in cold lysis buffer for 10 min. Total cell lysates were separated by SDS-PAGE, transferred to nitrocellulose membrane, and reacted with antibodies to PTD-poMx1. The blot was stripped and re-probed with anti-β-actin antibody as a loading control. **d** The stability of PTD-poMx1. ST or 3D4/21 cells were treated with 80 μg/ml PTD-poMx1 for 6 h at 37 °C. After washing three times with PBS, cells in DMEM free from PTD-poMx1 were incubated for an additional 4, 8, 12, 16, and 20 h. Intracellular PTD-poMx1 in the cell lysates was detected using anti-poMx1 mAb as previously above

Our previous results have shown that PTD-poMx1 expressed in *E.coil* entered Vero cells after 5 h incubation and maintained for 20 h in the cytoplasm. However, the internalization and stability of PTD-poMx1 in porcine cell line are unknown even though PTD-poMx1 can inhibit CSFV replication in PK-15 cells. Some confusing details are as follows: i). how long the exogenous PTD-poMx1 enters the porcine line. ii) how long it can stay after entering the cells. To address these questions, a series of experiments were performed as described previously [17]. PTD-poMx1 was used at 80 μg/ml, a concentration that had already been shown to be non-toxic to PK-15 cells. First, the intracellular localization of PTD-poMx1 in ST or 3D4/21 cells was determined using immunofluorescence by confocal microscopy analysis. Fluorescent spots were detected in the cytoplasm after incubation for 6 h and increased along with incubation time (Fig. 1a, b), indicating that PTD-poMx1 could efficiently penetrate cell membranes within a few hours and localize in the cytoplasm. Second, the transduction efficiency of PTD-poMx1 was determined using anti-poMx1 mAb by Western blot analysis. As shown in Fig. 1c, PTD-poMx1 was detected in ST and 3D4/21 cells after 3 and 2 h, respectively, and more was detected along with incubation time. These results demonstrated that PTD-poMx1 entered both ST and 3D4/21 cells. Another Western blot analysis was performed to determine the stability of intracellular PTD-poMx1 in ST or 3D4/21 cells. As shown in Fig. 1d, PTD-poMx1 was detected in cell lysates of ST or 3D4/21 cells up to 16 h, but not at 20 h.

### PTD-poMx1 did not affect CSFV entry

**Fig. 2:**
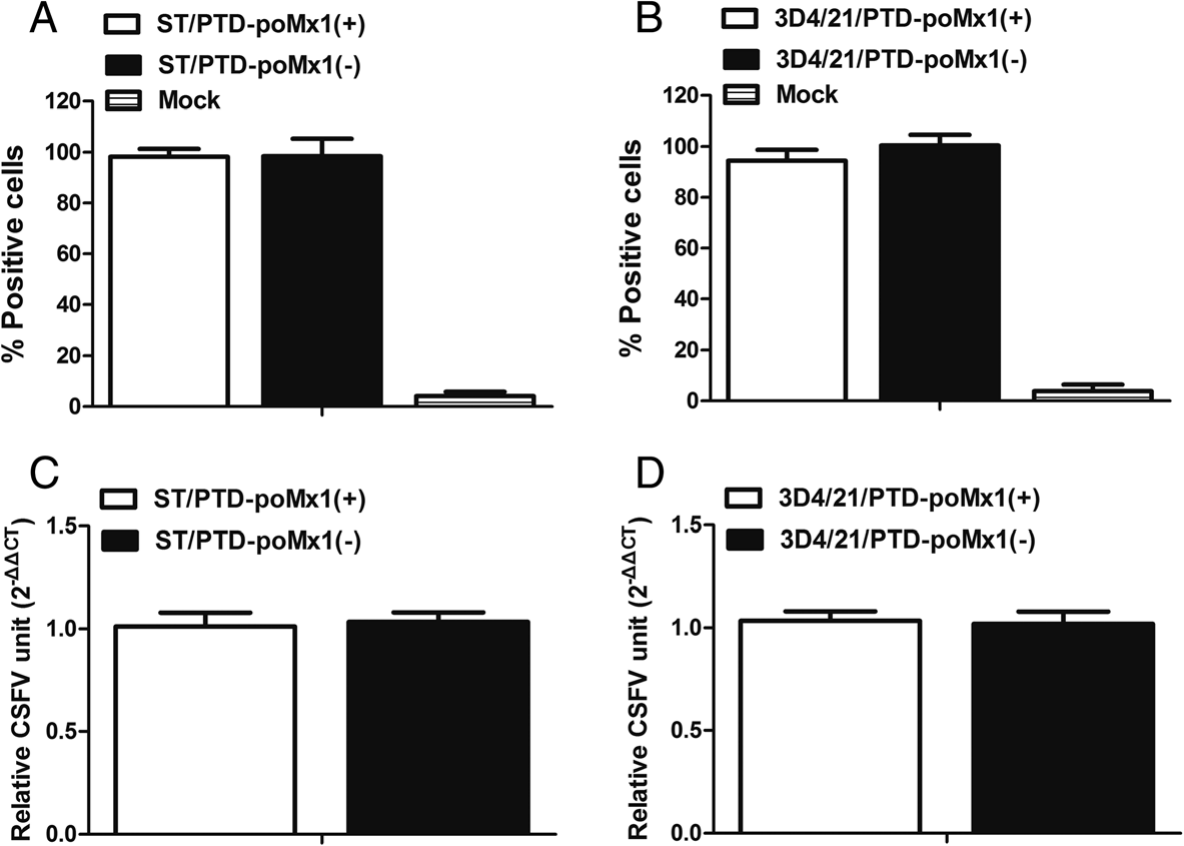
PTD-poMx1 did not inhibit CSFV entry. Virus binding to the surface of ST (**a**) and 3D4/21 (**b**) cells with or without PTD-poMx1 was determined using flow cytometry analysis. ST or 3D4/21 cells treated with or without 80 μg/ml PTD-poMx1 were inoculated with CSFV at a MOI of 5 for 1 h on ice. The infected cell suspensions were prepared with PBS containing 0.02 % EDTA, and incubated with the anti-E2 monoclonal antibody (WH303) for 1 h at 37 °C. Cells were washed three times with PBS and treated with FITC-labeled goat anti-mouse IgG (1:200) at 37 °C for 30 min. Fluorescent signals on the cell surface were detected and the percentage of positive cells was counted in a sample of 3 × 10^4^ cells. Untreated cells were used as a negative control. Virus uptake in the ST (**c**) or 3D4/21 (**d**) cell line with or without PTD-poMx1 was then determined using qRT-PCR. Cells were preincubated with 80 μg/ml PTD-poMx1 for 6 h and incubated with CSFV on ice for 1 h. Then, cells were washed three times with PBS and cultured for 2 h at 37 °C. Internalized viruses were quantified using qRT-PCR

Our previous studies showed that PTD-poMx1 did not affect VSV entry into Vero cells [17], but little is known about whether PTD-poMx1 affects adsorption, binding or uptake of CSFV during infection of porcine cells. To examine this more closely, flow cytometry was used to evaluate the process of adsorption and binding of CSFV-infected cells in the presence or absence of PTD-poMx1. The results showed that the percentage of fluorescent ST or 3D4/21 cells treated with PTD-poMx1 did not decrease significantly compared to the untreated cells (Fig. 2a, b), suggesting that treatment with PTD-poMx1 did not affect the specific entry of CSFV. Second, a qRT-PCR assay was performed to evaluate the uptake process. The results showed that the relative viral loads were similar between the PTD-poMx1-treated and untreated cells (Fig. 2c, d), suggesting PTD-poMx1 did not affect CSFV uptake.

### In vitro evaluation of antiviral activity of PTD-poMx1

**Fig. 3:**
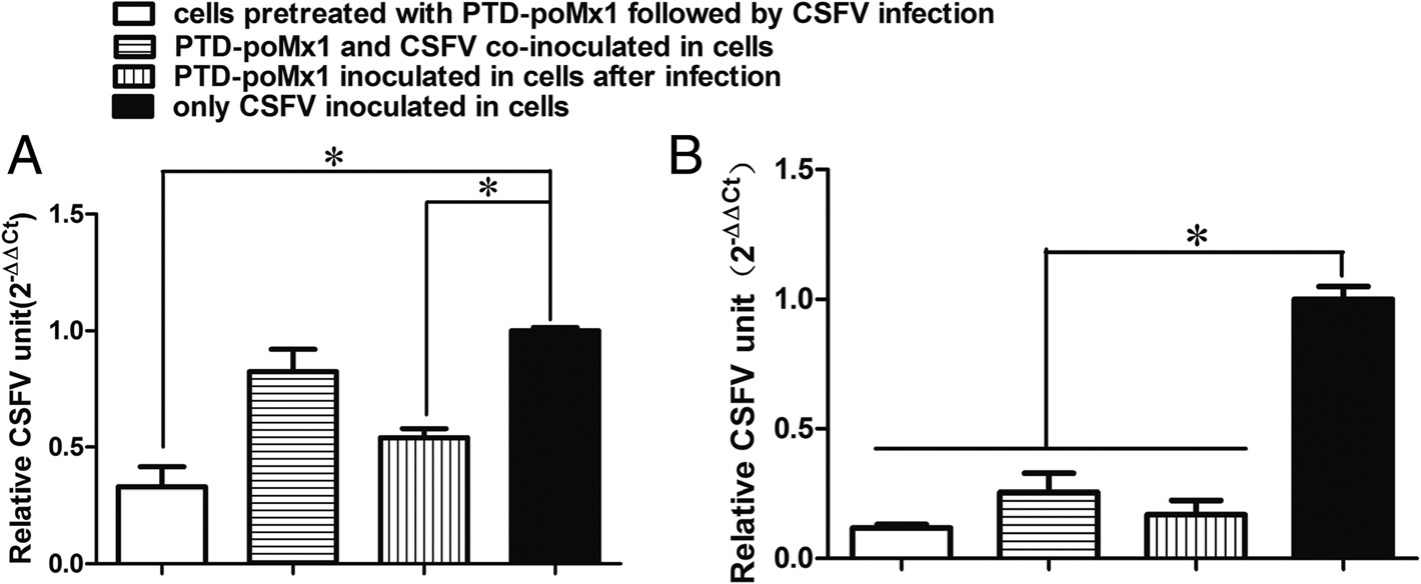
Antiviral activity in ST (**a**) or 3D4/21 (**b**) cells in response to three different PTD-poMx1 treatments. Cells were treated with 80 μg/ml PTD-poMx1 and 0.01 MOI CSFV as follows: (*i*) cells pretreated with PTD-poMx1, followed by CSFV infection; (*ii*) cells co-inoculated with PTD-poMx1 and CSFV; (*iii*) cells infected with CSFV, then treated with PTD-poMx1. At 48 hpi, virus was released from cells by freezing and thawing three times, and qRT-PCR was performed to evaluate viral RNA load in vitro. Experiments were performed in triplicate and data are shown as mean ± S.D. Statistical significance is indicated as *(*P* < 0.05)

Since PTD-poMx1 did not affect virus binding and uptake, it is possible that PTD-poMx1 manifests the anti-viral activity after CSFV entry. To test this hypothesis, we conducted three series experiments by infecting cells with CSFV at MOI of 0.01 treating with PTD-poMx1 at different time point: (i) cells were pretreated with PTD-poMx1, followed by CSFV infection; (ii) cells were treated with PTD-poMx1 and infected with CSFV simultaneously; (iii) cells were infected with CSFV first, and then treated with PTD-poMx1. Viral RNA loads were evaluated by qRT-PCR as described above. As shown in Fig. 3, viral RNA loads in cells treated with PTD-poMx1 either before or after CSFV infection were significantly decreased (*p* < 0.05) compared to those in CSFV-infected cells without PTD-poMx1 treatment. No significant change in RNA level was observed in the cells that had been treated with PTD-poMx1 and infected with CSFV simultaneously, possibly because the co-inoculation time was too short to transduce enough PTD-poMx1 into ST cells to inhibit CSFV replication (Fig. 3). In addition, there was no significant difference between the cells treated with PTD-poMx1 before and after infection, suggesting that PTD-poMX1 inhibited CSFV replication after entry.

### In vivo evaluation of antiviral activity of PTD-poMx1 in *domestic pigs*

**Fig. 4:**
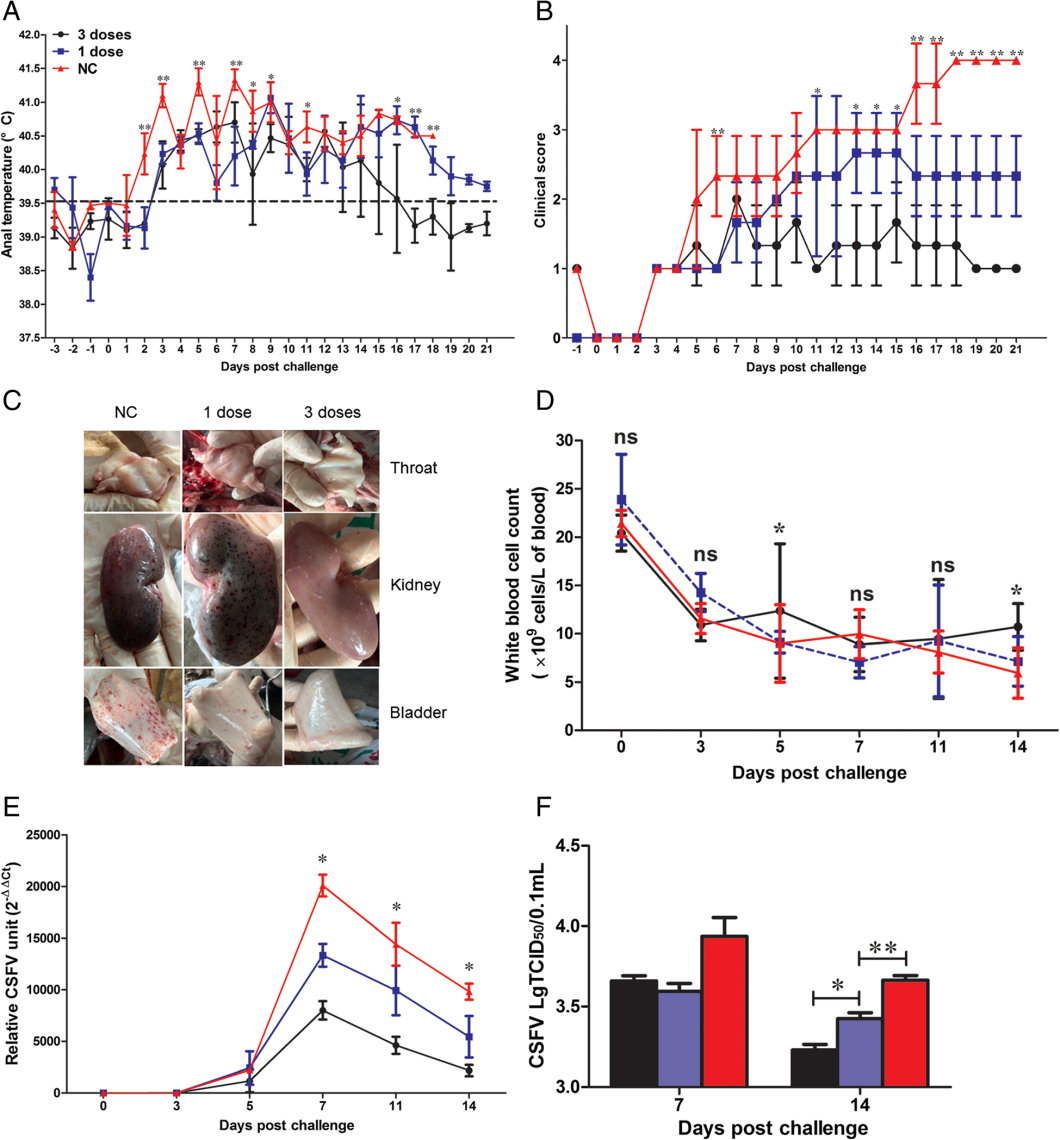
In vivo antiviral activity of PTD-poMx1 in pigs. Nine four-week-old specific-pathogen-free Large White pigs were randomly assigned to three groups of three pigs. All animals were oro-nasally challenged with the 10^5^TCID_50_ Shimen strain of CSFV. On the first day post challenge (1dpc), pigs in 1 dose group and 3 doses group were injected 1 mg PTD-poMx1/dose via ear vein, while those in the negative control group (NC group) received normal saline. Pigs in 3 doses group were injected two more times with 1 mg PTD-poMx1 at 3 and 5 dpc. Clinical parameters monitored in experimental groups: **a** Rectal temperature; **b** Clinical score; **c** Pathological lesion; **d** White blood cell count; **e** Viral load in sera using qRT-PCR; **f** Progeny viruses in sera using a TCID_50_ assay. All data were expressed as the mean (±S.D) of results obtained for the 3 pigs in each group within 3 weeks. Statistical significance was indicated as not significant (ns) (*P* > 0.05), *(*P* < 0.05) and **(*P* < 0.01)

Animal experiments were conducted to assess the antiviral activity of PTD-poMx1 in vivo*,* and three groups of pigs were used. Two experimental groups (1 dose and 3 doses groups) and the negative control group (NC group) were compared after CSFV challenge. First, as shown in Fig. 4a, the mean rectal temperature of pigs injected with either 1 dose or 3 doses was lower than that of untreated pigs at 2, 3, 5, and 7 dpc (*p* < 0.01). 3 doses group exhibited a significant decrease in rectal temperature at 14 dpc and thereafter (*p* < 0.05) when compared to group of 1 dose. By 16 dpc, the mean rectal temperature of any pig injected with 3 doses group became normal, while any pig received only 1 dose group still showed fever at 21 dpc. Second, all three groups were assessed based on the development of clinical signs such as loss weight gain, anorexia, conjunctivitis, constipation, abdominal petechiae, nervous disorders and prostration. As shown in Fig. 4b, the mean score of pigs injected with either 1 dose or 3 doses group was lower than that of untreated pigs since 5 dpc (*p* < 0.05). 3 doses group had a significantly lower score than 1 dose group since 11 dpc. Untill 18 dpc, all the pigs of NC group had died, while any pig of 1 or 3 doses group was alive. Furthermore, the pathological lesions verified the clinic signs. As shown in Fig. 4c, NC group showed serious hemorrhage spots in some tissues including throat, kidney and bladder. However, 1 dose or 3 doses group had less hemorrhage spots along with more injected doses. Third, as shown in Fig. 4d, a higher WBC count was apparent at 5 and 14 dpc (*p* < 0.05) in 3 doses group when compared to that in 1 dose or NC group. Finally, viremia was measured using TCID_50_ and qRT-PCR assays. As shown in Fig. 4e, we observed the increasing viral RNA loads until 7 dpc in all groups, followed by a decrease. The viral RNA load in NC group was higher than that in other groups since 5 dpc (*p* < 0.05). Moreover, at 7, 11 and 14 dpc, there were significant differences between viral RNA loads in 1 dose and 3 doses groups (*p* < 0.05). Also, a TCID_50_ assay was performed to quantify virus progeny. The trend was similar to that described earlier using qRT-PCR data (Fig. 4f). The mean viral titer of treated pigs was reduced at 14 dpc when compared to untreated pigs (*p* <0.01). Moreover, the mean viral titer of 3 doses group was significantly less than that of 1 dose group (*p* < 0.05). Overall, the resulting data above suggested that treatment with PTD-poMx1 could reduce viral loads and the duration of viremia depending on the number of doses.

**Fig. 5:**
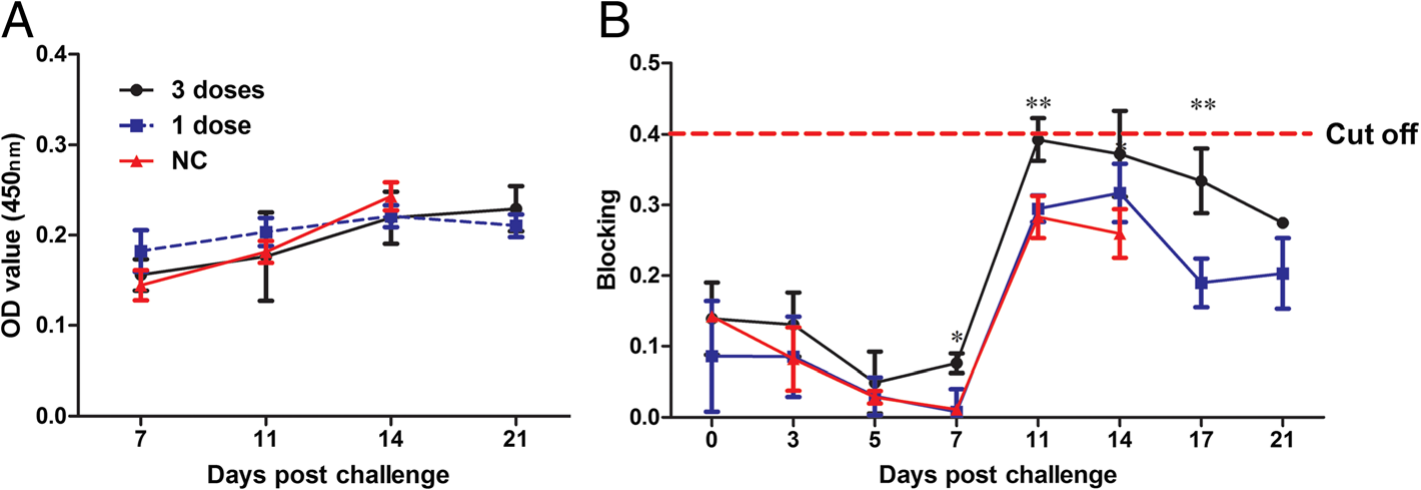
Specific antibody detected in sera using ELISA assay. **a** Antibody to PTD-poMx1; **b** Antibody to CSFV E2. All data were expressed as the mean (±S.D) of results obtained for the 3 pigs in each group within 3 weeks. Statistical significance was indicated as *(*P* < 0.05) and **(*P* < 0.01)

An ELISA assay was performed to assess the negative immune response to PTD-poMx1 in the pigs. As shown in Fig. 5a, no pigs injected either 1 dose or 3 doses produced specific antibodies against poMx1. Even though all the pigs of NC group died at 21 dpc, we further traced the antibody titer of the treated pigs of 1 dose and 3 doses groups. The results showed that the treated pigs were not induced immune response to PTD-poMx1, suggesting that PTD-poMx1 degraded quickly and did not induce an immune response. Another ELISA assay was performed to assess whether PTD-poMx1 could help the treated pigs to induce antibodies to CSFV E2. As shown in Fig. 5b, almost no pigs produced the positive humoral response against CSFV and the antibody titers were low below cut off value. Intriguingly, the antibody titer of the treated pigs were higher than that of the untreated pigs and the treatment of 3 doses clearly increased the antibody level.

## Discussion

This is the first report that poMx1 fusion protein can reduce CSF infection in vivo, suggesting a preliminary therapeutic treatment against CSF infection. Previous studies of interferon-induced Mx proteins and their antiviral activities have typically relied on in vitro models. For example, human MxA inhibits influenza virus in human A459 cells [30, 31] and CSFV in both PK-15 cells and swine fetal fibroblasts (PEF) [32]. Additionally, VSV replication can be inhibited by either porcine Mx1 in Vero cells [17] or porcine Mx2 in NIH3T3 cells [33, 34]. Our previous results have shown that overexpressing fusion protein EGFP-poMx1 and PTD-poMx1 both inhibited CSFV replication in PK-15 cells in a dose-dependent manner [21]. However, many details need to be further studied for elaborating how the exogenous PTD-poMx1 enters the porcine line, its stability after entering and where PTD-poMx1 interferes with viral replication in viral life cycle. To clarify the above questions, we used ST and 3D4/21 cells to further investigate the antiviral details. Our resulting data illuminated these perplexing problems as follows: (i) PTD-poMx1 transduced into porcine cells within 3 h, then localized in the cytoplasm, and was stable for 16 h. Even though PTD-poMx1 transduced into PK-15 cells more quickly (1 h), it degraded within 12 h (data not shown). These results indicated that the internalization efficiency of PTD-poMx1 is different among some cell lines. (ii) PTD-poMx1 did not affect CSFV entry, suggesting neither did it compromise cell surface receptors nor hinder CSFV absorption and uptake. (iii) qRT-PCR results showed that the viral titer was similar regardless of the treatment, which suggested that poMx1 inhibited CSFV replication post CSFV entry and the antiviral mechanism by which poMx1 inhibited CSFV replication may involve direct interaction with viral proteins but cellular proteins. This conflicted with a previous report that poMx1 inhibited influenza A virus replication by affecting the cellular early endosome [20]. Previous studies have revealed the antiviral mechanisms of human MxA and murine Mx1. However, the antiviral mechanism of porcine Mx1 against CSFV is unknown to date. Interaction with viral nucleoprotein (NP) is the most likely common pathway for MxA to perform its antiviral function against RNA viruses, in particular for influenza A virus [35, 36]. Based on their nucleotide and amino acid sequences, porcine Mx1 and human MxA (huMxA) are closely related (78 %) [13], suggesting that they have similar functions. Therefore, interaction of CSFV structural or nonstructural proteins with poMx1 might contribute to the antiviral function and need to be further identified through a series of experiments. In addition, another qRT-PCR assay was performed to evaluate the longevity of the antiviral activity of PTD-poMx1 in ST and 3D4/21 cells, which proves to be a function of time. Significant differences of viral genome copies were shown between treated and untreated cells at 24, 48 and 72 hpi, which agrees with the results obtained in PK-15 cells (data not shown).

CSF is an epidemic disease that threatens the pig industry worldwide, and particularly in China. Although C strain is still considered one of the best CSFV vaccines, it cannot be used as a prophylactic vaccine in the European Union (EU) because it is impossible to serologically differentiate vaccinated from infected pigs [5]. Fortunately, the CP7_E2alf marker vaccine is currently used in some EU countries as a substitute for the C strain [6, 8] but its immune effect is under evaluation. Nevertheless, any vaccine needs time to take effect [37] and pigs can still be infected during the period between vaccination and protection. Therefore, it is necessary to develop other antiviral strategies to control CSF. Additionally, despite a compulsory vaccination policy in China since 2007, there are still sporadic and endemic outbreaks in many areas [38]. As an alternative to mass culling, other measures, especially the use of antiviral agents, have been proposed [39–41]. Unfortunately, in China almost no effective strategies are available to control CSF transmission except for vaccination of uninfected pigs and antibiotic therapy in infected pigs. Moreover, antibiotics cannot effectively control the secondary bacterial infection because of drug resistance. Some inhibitors such as BPIP (5-[(4-Bromophenyl)methyl]- 2-phenyl-5H-imidazo[4,5-c]pyridine) ([41, 42] and BBP (2,6-Bis(benzimidazol-2-yl)pyridine) [9], exhibit anti-CSFV activities because they target viral RdRp and can significantly reduce viremia and viral load in infected pigs. Although they are used in the EU, it is not known when these drugs will be authorized for use in China.

Based on these considerations above and *in intro* experiments, we tested whether PTD-poMx1 could be used in infected pigs for CSF control as an antiviral agent. Our data demonstrated that PTD-poMx1 could reduce CSFV infection in domestic pigs. Fig. 5 showed that the CSFV-challenged pigs treated with PTD-poMx1 clearly demonstrated attenuated clinical signs and lower viral RNA loads from 5 to 21 dpc. The data also showed that PTD-poMx1 of 3 doses, as opposed to 1 dose, conferred stronger antiviral activity and is associated with lighter clinical signs and lower viral loads although viremia was detected in these pigs until 14 dpc. We speculate that a dose of higher concentration and/or an increasing number of doses will result in a stronger antiviral effect and more rapid decrease of viremia. Interestingly, the anti-poMx1 antibodies of the treated pigs were not detected in the treated pigs until 21 dpc, suggesting that PTD-poMx1 inhibited CSF replication early in infection and rapidly degraded in the pig’s body. In addition, the humoral response of the treated and untreated pigs were almost negative and finally the protection antibodies were not induced. These results were similar with previous report that only challenge pigs can’t be induced the positive immune response (ref). However, the antibody level in the treatment of 3 doses clearly increased, suggesting the treatment of poMx1 may induce a positive immune response that needs to be further testified.

## Conclusions

In summary, we demonstrated the anti-CSFV activity of PTD-poMx1 in vitro and in vivo. To the best of our knowledge, this is the first report that PTD-poMx1 inhibits CSFV infection in vivo. The results showed that treatment with PTD-poMx1 alleviated symptoms and viral loads in infected pigs. These data support and extend previous studies, and indicate that PTD-poMx1 may be an effective therapy against CSF infection in the future.
